# Dolutegravir Discontinuation for Neuropsychiatric Symptoms in People Living with HIV and Their Outcomes after Treatment Change: A Pharmacogenetic Study

**DOI:** 10.3390/metabo12121202

**Published:** 2022-12-01

**Authors:** Jessica Cusato, Alberto Borghetti, Elisabetta Teti, Maurizio Milesi, Maria Cristina Tettoni, Stefano Bonora, Mattia Trunfio, Antonio D’Avolio, Mirko Compagno, Simona Di Giambenedetto, Giovanni Di Perri, Andrea Calcagno

**Affiliations:** 1 Laboratory of Clinical Pharmacology and Pharmacogenetics, Department of Medical Sciences, University of Turin, 10149 Turin, Italy; 2 Institute of Clinical Infectious Diseases, Catholic University of Sacred Heart, 00168 Rome, Italy; 3 Department of Systems Medicine, Infectious Diseases Clinic, University Hospital “Tor Vergata”, 00133 Rome, Italy; 4 Unit of Infectious Diseases, Department of Medical Sciences, University of Turin, 10149 Turin, Italy

**Keywords:** psychiatric symptoms, depression, dolutegravir, pharmacogenetics, discontinuation

## Abstract

Neuropsychiatric symptoms have been reported in patients receiving dolutegravir, a known inhibitor of the renal and neuronal-expressed organic anion transporter 2 (encoded by *SLC22A2* gene). The effect of the genetic variant *SLC22A2* 808C>A on dolutegravir discontinuation was assessed and analyzed by real-time PCR. We enrolled 627 participants: CA/AA carriers showed a higher prevalence of pre-existing psychiatric comorbidities and use of antidepressants. After 27.9 months, 108 participants discontinued dolutegravir, 64 for neuropsychiatric symptoms. Patients with pre-existing psychiatric comorbidities were at higher risk of dolutegravir discontinuation, while patients carrying the *SLC22A2* CA/AA genotype were not. Combining the two variables, an opposite effect of *SLC22A2* variants according to pre-existing psychiatric disorders was observed. Using multivariate Cox models, the combined variable pre-existing psychiatric comorbidities/*SLC22A2* variants and the use of non-tenofovir alafenamide containing antiretroviral regimens were predictors of dolutegravir discontinuation for neuropsychiatric symptoms. Within 30 days, the majority of participants had a complete resolution of symptoms (61.8%), while 32.7% and 5.5% had partial or no change after dolutegravir discontinuation, respectively. Discontinuation of dolutegravir for neuropsychiatric symptoms was not uncommon and more frequent in participants with pre-existing psychiatric disorders. We described an interaction between *SLC22A2* genetic variant and psychiatric comorbidities. In 38.2% of patients, a complete neuropsychiatric symptoms resolution was not observed after dolutegravir discontinuation suggesting the involvement of additional factors.

## 1. Introduction

The antiviral efficacy of modern combination antiretroviral therapies (cART) is excellent, with more than 90% of patients suppressing HIV RNA within one year. Recently introduced antiretrovirals (ARVs) have shown a favorable tolerability profile: unboosted integrase strand transfer inhibitors (INSTI) have shown high rates of efficacy and limited drug-associated side effects [1]. Second-generation INSTI, namely dolutegravir (DTG) and bictegravir (BIC), have been associated with neuropsychiatric side effects and weight gain [2,3]. Data on DTG are more abundant but discordant. Sleep abnormalities, headache and neuropsychiatric intolerance have been observed in several observational cohorts but very uncommonly in randomized clinical trial [4,5,6,7]. In the recent ADVANCE trial, almost no patients experienced insomnia or other neuropsychiatric side effects (NPS) [8]. Apart from different inclusion and exclusion criteria, these discrepancies may reveal ethnic and genetic factors potentially associated with NPS. Older age, female sex, abacavir coadministration, and DTG plasma exposure have been identified as risk factors and they vary significantly among studies [9,10]. The evidence of higher cumulative incidence of neuropsychiatric side effects in Japanese patients carrying genetic variants in the uridine diphosphate-glucuronosyltransferase 1A1 (*UGT1A1*) encoding gene suggest a potential role for pharmacogenetics [11].

The drugs DTG and BIC are inhibitors of organic cation transporter 2 (OCT-2) enzyme with similar 50% inhibitory concentrations. The transporter OCT-2 is broadly expressed in the central nervous system (CNS) besides renal tubular cells [12]. This transporter enzyme has been localized in neuronal cell bodies, presynaptic membranes, and choroid plexus. Previous studies suggested that it may be involved in the transport of monoamine, dopamine, serotonin, histamine, creatinine, and choline [13,14]. The transporter OCT2 was found to be expressed in the limbic system and to be involved in anxiety- and depression-related behaviors in experimental animals. Additionally, genetic deletion of *SLC22A2* (the gene encoding for OCT-2) was associated with a significant reduction in brain tissue concentrations of norepinephrine and serotonin and affected long-term response to treatment with antidepressants [15]. Our group previously reported that *SLC22A2* 808 C>A variant and DTG plasma concentrations were associated with psychiatric symptoms in a cohort of treated HIV-positive patients [16].

For these reasons we conducted a study to evaluate the influence of the *SLC22A2* 808 C>A polymorphism on the incidence of DTG discontinuation in people living with HIV (PLWH) starting this antiretroviral.

## 2. Experimental Design

An observational retrospective/prospective pharmacogenetic study was conducted in three Italian outpatients’ clinics (one in Turin and two in Rome) between 2015 and 2020. The protocol was approved by the three local Ethics Committees. Inclusion criteria were: aged at least 18 years, HIV-positivity evaluated through Western-blot technique, one dose of DTG administration, and signed informed consent. No exclusion criteria were considered. All patients that had initiated DTG (retrospective, including those who had interrupted the drug) or those starting DTG after 2018 (prospective) were invited to participate. Patients were administered antidepressants, anxiolytics, and methadone/buprenorphine concomitant drugs. All participants signed written informed consent before study enrolment. Patients were followed according to clinical practice.

## 3. Procedure

Whole blood (3 mL, at the same time of another blood withdrawal of a visit planned for the routinary clinical practice) for pharmacogenetic analysis was collected and stored at −20 °C. It was subsequently dry ice-shipped to the Laboratory of Clinical Pharmacology and Pharmacogenetics of the University of Turin, Turin. Genomic DNA was extracted using a QIAamp whole Blood Mini Kit (Qiagen, Valencia, Santa Clarita, CA, USA) according to the manufacturer’s instructions. SLC22A2 808 (rs316019) C>A polymorphism (genetic variant leading to an amino acid change altering the function of SLC22A2) genotyping was conducted by real-time PCR-based allelic discrimination with the use of Taqman probes and Master Mix with nucleotides and Taq Polymerase (Thermofisher, Rodano, Milano, Italy) by using the CFX-96 real-time PCR (Biorad, Segrate, Milano, Italy).

The outcome of interest was DTG discontinuation for any cause and discontinuation for NPS. These symptoms were recorded in the medical records as described by physicians and in the study case report forms (CRFs) and were classified as headache, sleep disorders, anxiety, depression, psychotic symptoms, or others (including vertigo or confusion). Pre-existing psychiatric comorbidities were also extracted from medical records and study CRFs and classified as: depression, anxiety, psychotic, and substance abuse.

The sample size calculation estimated that enrolling 579 patients would allow the identification of a 30% effect of *SLC22A2* CA/CC variants on DTG discontinuation (with alpha of 0.05 and 0.90 power). Data are described using parametric tests. Kaplan-Meier curves with log-rank analysis and Cox proportional hazard models were used. The study ended in June 2020 and patients with at least 3 months of follow up and with an available *SLC22A2* genotype were included.

## 4. Results

### 4.1. Participants Characteristics

We enrolled 627 participants: their characteristics are reported in Table 1.

The majority of participants were male (439, 70%) aged 50.5 years (±11.3) and with a body mass index of 24.4 Kg/m^2^ (±4.1). Baseline CD4 cell count was 569 (±498) and 253 (40.3%) participants were already on treatment and with serum HIV RNA < 50 copies/mL at DTG initiation. Chronic hepatitis was recorded in 193 participants [namely HCV (n = 133), HBV (n = 35), or both (n = 25)]. Psychiatric comorbidities were observed in 100 participants (15.9%), while antidepressants, anxiolytics, and methadone/buprenorphine were reported in 46 (7.3%), 42 (6.7%), and 28 (4.5%) participants.

The majority of study participants were receiving three ARVs (377, 60.1%) with a significant proportion of participants receiving two-drug regimens [(“2DR”) 230, 36.7%]. Besides DTG, lamivudine/emtricitabine (355, 94.2%), abacavir (169, 44.8%), tenofovir alafenamide (105, 27.9%), and tenofovir disoproxil fumarate (86, 22.8%) were the ARV more commonly used in three-drug combinations. Patients on 2DR 235 were co-administered with lamivudine (95, 41.3%), darunavir (51, 22.2%), atazanavir (46, 20%), or rilpivirine (32, 13.9%). Among participants receiving boosted protease inhibitors (bPIs), 68 (10.8%) were receiving ritonavir and 34 (5.4%) cobicistat.

**Figure 1:**
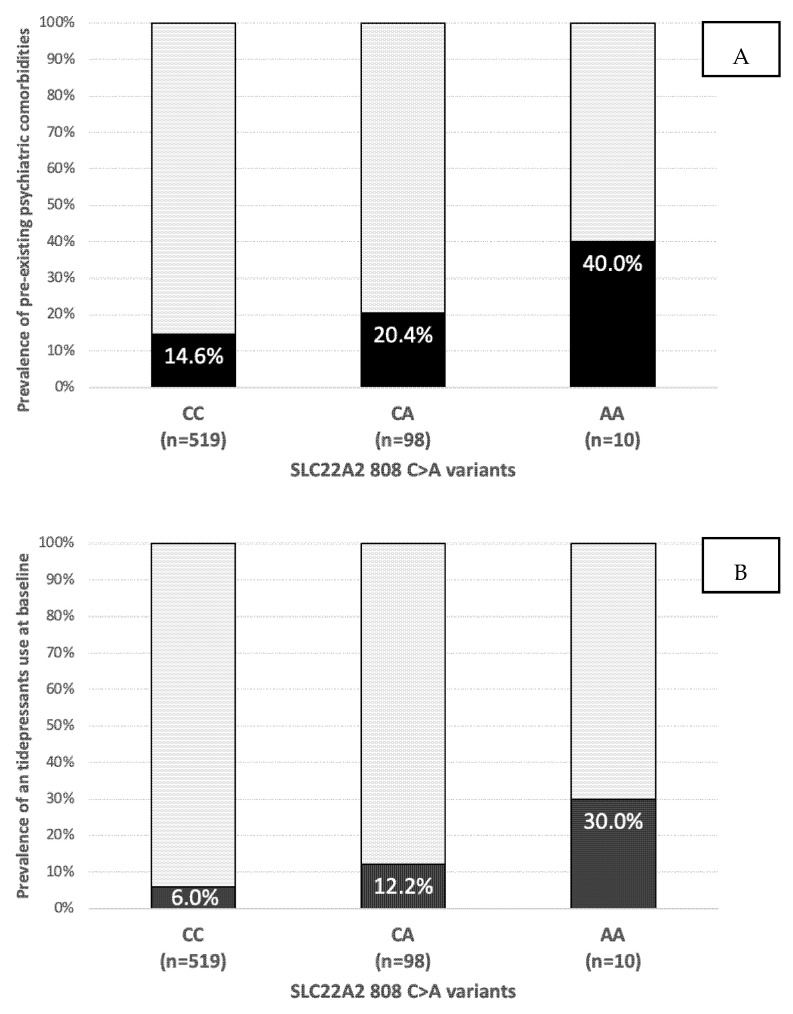
Prevalence of pre-existing psychiatric comorbidities (**A**) and antidepressant use (**B**) according to *SLC22A2* variant.

Pharmacogenetic analysis identified SLC22A2 808 CC (519, 82.8%), CA (98, 15.6%), and AA 10 (1.6%) variants and we grouped together CA/AA genotypes (n = 108, 17.2%). The population is not in Hardy-Weinberg equilibrium (chi squared = 4.39, the hypothesis that the observed and expected values are equivalent is rejected, calculated through the website https://wpcalc.com/en/equilibrium-hardy-weinberg/ (accessed on 30 November 2022)). The prevalence of pre-existing psychiatric comorbidities (14.6%, 20.4%, and 40%) and of antidepressant use (6%, 12.2%, and 30%) according to *SLC22A2* variants are depicted in Figure 1.

People with CA/AA genotype showed a higher prevalence of pre-existing psychiatric comorbidities (22.2% vs. 14.6%. *p* = 0.05, RR 1.66) and were more commonly receiving antidepressants (13.9 vs. 6.0%, *p* = 0.004, RR 2.53) as shown in Figure 1.

### 4.2. Incidence and Reasons for DTG Discontinuation

After a follow up of 27.9 months (±13.3), 108 participants (17.2%) discontinued DTG. The most commonly described reasons were NPS (64, 10.2%), simplification (10, 1.6%), and gastrointestinal side effects (8, 1.3%). Discontinuing participants reported the following NPS: sleep abnormalities (21, 19.4%), anxiety (18, 16.7%), headache (17, 15.7%), depression (12, 11.1%), psychosis (3, 2.8%), vertigo (2, 1.9%), and confusion (2, 1.9%). Patients with pre-existing depression (33.3% vs. 5.7%, *p* = 0.002, RR 8.2) and on antidepressants (30.7 vs. 8.4%, *p* = 0.037, RR 4.83) had a higher incidence of depression as a reason for discontinuation.

### 4.3. Factors Associated with DTG Discontinuation

Using bivariate log-rank analysis, the following variables were associated with DTG discontinuation: tenofovir disoproxil fumarate (TDF) (*p* = 0.049), antidepressants (*p* = 0.022), anxiolytics (*p* < 0.001) use, and pre-existing psychiatric co-morbidities (29.0 vs. 14.9%, *p* < 0.001). Using bivariate log-rank analysis, the following variables were associated with discontinuation for NPS: non-tenofovir alafeniamide (TAF) use (*p* = 0.044), antidepressant use (*p* = 0.023), anxiolytics use (*p* < 0.001), and pre-existing psychiatric co-morbidities (21.7 vs. 8.9%, *p* < 0.001, Supplementary Figure S1).

Given the observed interaction between *SLC22A2* variant and pre-existing psychiatric comorbidities, we subsequently separately analyzed variables associated with DTG discontinuation in participants without [*SLC22A2* CA/AA carriers (*p* = 0.050) and TDF use (*p* = 0.028)] and with [anxiolytic use (*p* = 0.054)] pre-existing psychiatric comorbidities. The probability of discontinuing DTG according to *SLC22A2* variants was different in participants without (higher in CA/AA carriers) and with (lower in CA/AA carriers) pre-existing psychiatric disorders.

**Figure 2:**
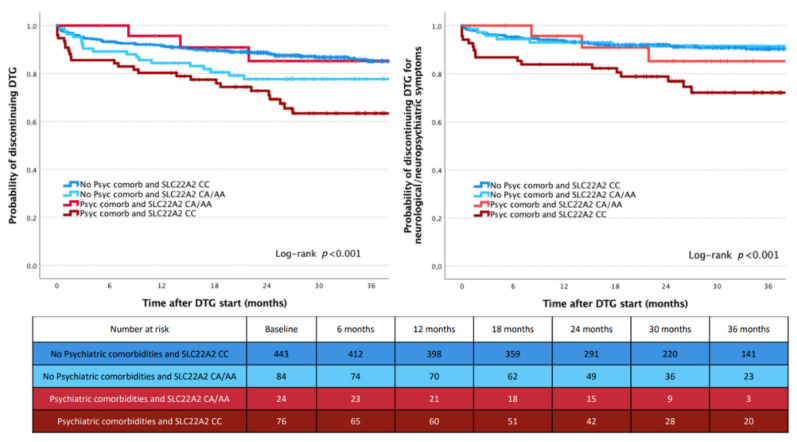
Risk of dolutegravir discontinuation for all causes (**left** panel) and for neuropsychiatric symptoms (**right** panel) according to the variable combining pre-existing psychiatric comorbidities and SLC22A2 variant.

We therefore generated a variable combining pre-existing psychiatric comorbidities (presence or absence) and *SLC22A2* genotypes (CC or CA/AA): this variable was significantly associated with the rate of discontinuation for any reason (Log-rank *p* < 0.001) and for NPS (Log-rank *p* < 0.001). Kaplan-Meier curves are shown in Figure 2.

Patients with pre-existing psychiatric comorbidities and carrying *SLC22A2* CC genotype had the highest risk of discontinuing DTG for any cause (26/76, 34.2%) and for NPS (17/68, 25%).

We observed no difference in the rates of discontinuation for any reason and for NPS according to the number of ARVs (dual vs. triple) or according to the treatment status (naive vs. experienced).

Using the multivariate Cox proportional hazard model (including age, gender, antidepressant, and anxiolytics use) we identified the use of TDF (*p* = 0.042, adjusted hazard ratio (aHR) 1.630, 95% interval of confidence (CI) 1.019–2.609) and the combined variable pre-existing psychiatric comorbidities/*SLC22A2* variants (*p* = 0.001, aHR 1.329, 95%CI 1.116- 1.582) as independent predictors of DTG discontinuation. At the multivariate Cox proportional hazard model (including age, gender, TAF-coadministration, antidepressant, and anxiolytics use) we identified the combined variable pre-existing psychiatric comorbidities/*SLC22A2* variants (*p* = 0.015, aHR 1.331, 95%CI 1.058–1.673) and the use of non-TAF containing antiretroviral regimens (*p* = 0.036, aHR 2.674, 95%CI 1.066–6.704) as independent predictors of DTG discontinuation for NPS.

### 4.4. Follow Up of Participants after DTG Discontinuation

**Figure 3:**
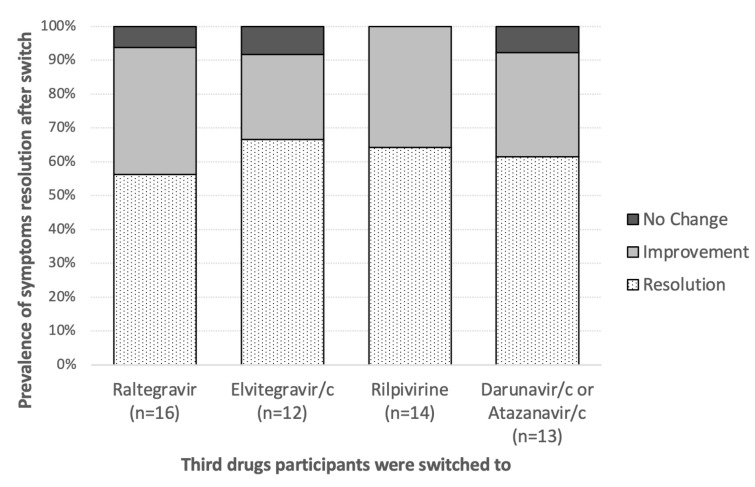
Prevalence of neuropsychiatric symptom resolution after dolutegravir discontinuation according to the antiretrovirals patients were switched to.

The most commonly used ARVs patients switched to were raltegravir (28, 30.1%), elvitegravir/cobicistat (26, 28%), protease inhibitors (atazanavir or darunavir, 25, 26.9%) and rilpivirine (14, 15.1%). We have detailed information on the outcome of 55 participants out of 64 discontinuing DTG for NPS: within 30 days the majority had a complete resolution of side effects (34, 61.8%) while 18 (32.7%) and 3 (5.5%) had partial or no resolution of symptoms, respectively. No significant change in the outcome of NPS was observed according to the drugs participants were switched to (*p* > 0.05) (Figure 3). On the contrary we observed that certain symptoms had a better outcome: sleep disturbances (80.0 vs. 48.9%, *p* = 0.03) and headache (82.3 vs. 50%, *p* = 0.024) showed a complete resolution in the majority of participants.

## 5. Discussion

In this retrospective/prospective study we assessed the incidence of DTG discontinuation according to several variables including pharmacogenetic biomarkers. We identified a significantly higher rate of DTG withdrawal in participants previously diagnosed with psychiatric comorbidities.

After more than 2 years, we observed that 17.2% of study participants stopped DTG but NPS were reported by 10.2% of them. This incidence is higher than the one observed in the majority of cohorts that reported rates between 1.7% and 5.6%, while three studies had similar discontinuation rates (8.2%, 9.9% and 11%) [17,18,19,20,21,22,23,24]. It should be noted that the definitions of NPS varied among studies and that our trial was focused on treatment discontinuation and may have, potentially, enhanced physicians’ reaction to patients complaints. Data from an observation study support this hypothesis: NPS effects were observed in 11% of study participants but led to DTG discontinuation only in 1% of them [19]. These incidence rates are in striking contrast with randomized trials where discontinuation rates for NPS were very uncommon [8].

We tested whether the already recognized risk factors applied to our study population. We found no clear effect of gender, age, or abacavir coadministration. Unfortunately, we did not collect DTG trough concentrations and therefore we are not able to confirm or confute the potential role of higher exposure on the risk of NPs [11,25,26]. Age, however, seems to be mildly associated with higher drug exposure and thus potentially linking the previously observed slightly higher risk in elderly patients (that are usually underrepresented in clinical trials) [27]. We did not observe a higher risk in patients receiving 2DR nor in those receiving abacavir; however, at univariate analysis we identified TDF as a risk factor for DTG discontinuation for all causes and non-TAF containing regimens for NPs. The first may be explained by a widespread avoidance of TDF in ageing patients that may have led to treatment switched for renal or bone toxicity and/or simplification. On the contrary, nonTAF-containing regimens are heterogenous and we were not able to identify a safety signal in other coadministered ARVs.

The major determinant of DTG discontinuation for all causes and for NPs was the presence of pre-existing psychiatric comorbidities. This is in contrast with what was reported by a large cohort study in the US and it may be peculiar of the management of PLWH in Italy. In our setting, infectious diseases consultants are the physicians in charge of patients’ treatments and this may lead to the preventive discontinuation of drugs perceived to be detrimental despite mild or moderate symptoms [28]. We were able to analyze the effect of specific psychiatric medication classes and found that anxiolytics were associated with a highest risk (and they were retained in the multivariate model). Unfortunately, we are not able to discriminate the potential pharmacodynamic interaction of these drugs with DTG or the effect of the specific underlying psychiatric comorbidity on the risk of interrupting DTG. Yet our Cox proportional hazard model identified psychiatric disorders as the only variable (besides nonTAF-containing regimens use) independently associated with the risk of discontinuing for NPS. Depression, among such conditions, seemed to be associated with an eight times higher risk of stopping DTG for depressive symptoms.

The primary objective of our study was assessing the impact of genetic variant in OCT2 encoding gene on the rate of DTG discontinuation. The hypothesis was built upon data obtained in vitro or in experimental animals and from a previous cross-sectional study from our group. In the latter NPS, assessed by tailored questionnaires, *SLC22A2* CA/AA carriers had a higher risk of having neuropsychiatric symptoms; we also reported a higher prevalence of psychiatric comorbidities in patients with such variants [16]. We confirm here that *SLC22A2* CA/AA carriers had a significantly higher risk of psychiatric disorders and of being treated with antidepressants. This is a novel finding since in recent genome-wide association studies no strong signal was observed for solute carrier transporters. Interestingly, *SLC6A4* and *SLC6A15* gene variants have been associated with age at depression onset [29,30]. Despite this lack of specific data on *SLC22A2* variants on the risk of depressive disorders, gene-diseases association biobanks suggest they may have the second highest association strength (after SLC6A4) [31]. The transporter OCT2 is a low-affinity carrier for a variety of physiological compounds including catecholamine, serotonin and choline neurotransmitters and it takes part in the postsynaptic reuptake of the extraneuronal neurotransmitters (including catecholamines). In this context, OCT2 may influence the response to stress (as observed in rodents). By decreasing extracellular monoamine concentrations, OCT2 inhibits the corticosterone release driven by the hypothalamic–pituitary– adrenocortical system, and this reduces stress and depression-like behaviors [32]. Genetic polymorphisms in SLC22A2 have already been demonstrated to influence substrate transport activity. The SNP rs316019 (808 A>C) is the most common variant of SLC22A2 with a frequency as high as 15% or more in several populations. The genotype SLC22A2 808 AA clears metformin (a known OCT2 substrate) from circulation at a much reduced level compared to CC carriers [33]. The hypothesis was therefore that patients with the CA/AA genotype receiving DTG (further inhibiting OCT2 activity) may have a higher risk of NPS. Unfortunately, given the relatively low number of CA/AA carriers, and the interaction with psychiatric comorbidities, we were not able to confirm this hypothesis. However, we demonstrated a differential effect of *SLC22A2* variants in participants with and without pre-existing psychiatric disorders; it was associated with a higher risk of DTG discontinuation (for any cause) in patients free of psychiatric comorbidities and with a lower rate of DTG interruption in the other group. This differential effect may be difficult to explain but compensatory mechanisms (in patients with chronic hypoactivity of OCT2) or drug-neurotransmitter interactions (as supported by the high prevalence of antidepressants in psychiatric participants) are working hypotheses. Interestingly patients with pre-existing psychiatric comorbidities and carrying loss of function genes in SLCA22A2 presented a risk for discontinuing DTG for NPs similar to patients lacking such comorbidities. Since OCT2 inhibitors have been developed in the past as antidepressants, a potentially beneficial effect of DTG may be hypothesized. A recent study showed that dasatinib (a well-known OCT2-inhibitor) reduced oxaliplatin-induced peripheral neuropathy [34].

We finally observed the outcome of patients stopping DTG for NPS. We describe here three not yet observed findings. We observed no significant differential effect according to the new ARVs patients were administered with thus supporting the use of drugs from the same class (such as INSTI) in case of this uncommon side effect. Second, we observed that only 61.8% of participants had a complete resolution of symptoms after discontinuing the drug. This may suggest that the causative role of DTG may be partial and pre-existing conditions could have a larger impact on the incidence of NPS. This is further reinforced by our third observation that headache and sleep abnormalities were the symptoms with the highest chance of being cleared once DTG was stopped, thus supporting some more specific hallmarks of DTG neuropsychological toxicity.

## 6. Conclusions

In conclusion, we observed that 10.2% of patients starting DTG discontinued the drug for neurological/neuropsychological side effects and that those with pre-existing psychiatric comorbidities and with genetic variants in *SLC22A2* gene (encoding for OCT2) were at higher risk of such adverse outcomes. We also reported here that the single nucleotide polymorphisms we studied (*SLC22A2* 808 C>A) was unevenly distributed among people living with HIV diagnosed with psychiatric disorders, suggesting a potential genetic/disease interaction. We finally reported here that in 38.2% of patients, a complete resolution of NPS was not observed after DTG discontinuation, suggesting the involvement of additional factors.

## Figures and Tables

**Table 1:** Baseline characteristics of study participants.

	Number (%) or Average (±Standard Deviation)
European ancestry	300 (84%)
Male sex at birth	439 (70%)
Age (years)	50.5 (±11.3)
BMI (Kg/m^2^)	24.4 (±4.1)
CD4 cell count (cell/mm^3^)	569 (±498)
Patients on treatment and with HIV RNA < 50	253 (40.3)
Positive serology for:	
HCV	133 (21.7%)
HBV	35 (5.6%)
HCV/HBV coinfected patients	25 (4.0%)
Pre-existing psychiatric comorbidities	100 (15.9%)
Antidepressant use	46 (7.3%)
Anxiolytics use	42 (6.7%)
Methadone/buprenorphine use	28 (4.5%)
Three-drug based therapies	377 (60.1%)
Lamivudine/emtricitabine	355 (94.2%)
Abacavir	169 (44.8%)
Tenofovir disoproxil fumarate	86 (22.8%)
Tenofovir alafenamide	105 (27.9%)
Two-drug based therapies	230 (36.7%)
Lamivudine	95 (41.3%)
Darunavir/c	51 (22.2%)
Atazanavir or atazanavir/c	46 (20%)
Rilpivirine	32 (13.9%)
SLC22A2 808 C>A carriers:	
CC	519 (82.8%)
CA	98 (15.6%)
AA	10 (1.6%)

## Data Availability

Data are available on request by the corresponding author.

## Supplementary Materials

The following supporting information can be downloaded at: https://www.mdpi.com/article/10.3390/metabo12121202/s1. Figure S1. Risk of dolutegravir discontinuation for all causes (left panel) and for neuropsychiatric symptoms (right panel) according to pre-existing psychiatric comorbidities. Log-rank *p* compares all 4 strata risk over time.
