# Supplementary figures and images for: Dolutegravir Discontinuation for Neuropsychiatric Symptoms in People Living with HIV and Their Outcomes after Treatment Change: A Pharmacogenetic Study
Source: Metabolites. 2022 Dec 1;12(12):1202. doi: 10.3390/metabo12121202 (PMC9781993; doi:10.3390/metabo12121202)

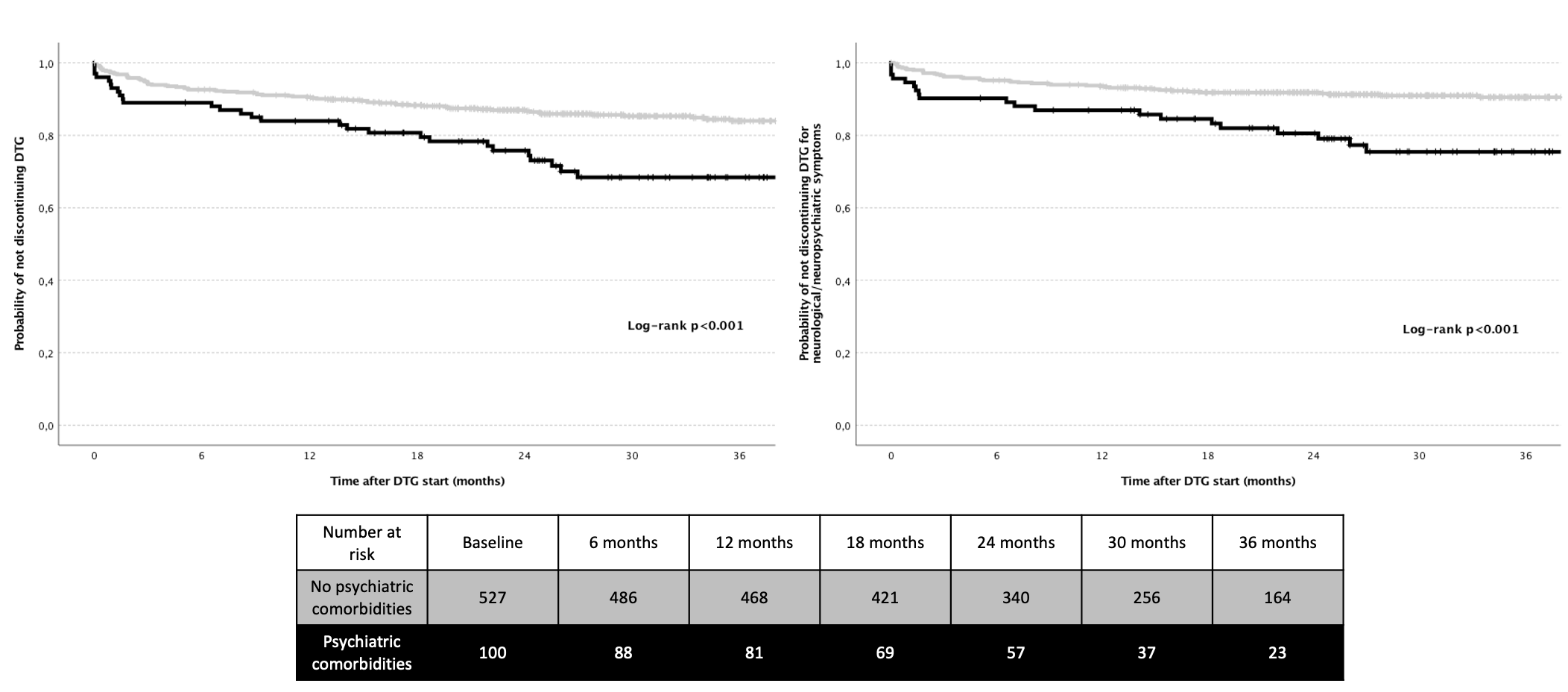

Supplement: Supplementary file 1 [file metabolites-12-01202-s001.zip › DOLUOCT2_SuppFigure S1.png]
